# Relative contribution of shoot and ear photosynthesis to grain filling in wheat under good agronomical conditions assessed by differential organ δ^13^C

**DOI:** 10.1093/jxb/eru298

**Published:** 2014-07-22

**Authors:** Rut Sanchez-Bragado, Gemma Molero, Matthew P. Reynolds, Jose Luis Araus

**Affiliations:** ^1^Unitat de Fisiología Vegetal, Facultat de Biologia, Universitat de Barcelona, Diagonal 645, Spain; ^2^International Maize and Wheat Improvement Center (CIMMYT), El Batán, Texcoco CP 56130, Mexico

**Keywords:** Carbon isotope composition, ear, flag leaf, grain filling, photosynthesis, shoot.

## Abstract

During grain filling in C3 cereals, the shoot (particularly the flag leaf) and the ear are believed to play major roles as sources of assimilates. However, both the cost and the intrusive nature of most of the methodologies available to investigate this have prevented conclusive results being obtained. This study compared the carbon isotope composition (δ^13^C) in its natural abundance in mature kernels with the δ^13^C of the water-soluble fraction of the peduncle, glumes, and awns to assess the relative contribution of the shoot (understood as the whole set of photosynthetic organs below the peduncle) and ear to grain filling in a set of highly productive wheat lines from the International Maize and Wheat Improvement Center, Mexico, under good agronomic conditions. In overall terms, the contribution of the ear was greater in comparison with that of the shoot. The specific contribution of the flag leaf blade to grain filling was also assessed by comparing the δ^13^C of grains with the δ^13^C of the water-soluble fraction of the flag leaf and the awns. The contribution of the flag leaf was minor, ranging between 3 and 18%. Complementary analyses performed such as gas-exchange rates and the accumulated water-soluble carbohydrates in both organs and light intercepted by the canopy at different strata suggested that the ear has a photosynthetic capacity at least comparable to that of the flag leaf. In this sense, selection for a higher contribution of ear photosynthesis to grain yield in breeding programmes could be addressed with the use of stable isotopes.

## Summary

We present a non-intrusive method to quantify the relative contribution of different photosynthetic organs to grain filling in cereals based on their discrimination among isotopes of carbon fixed, highlighting the key role of the ear.

## Introduction

The United Nations prediction for 2050 is that the world’s human population will reach 9.3 billion (Food and Agriculture Organization of the United Nations, 2013). The challenge to accommodate this world population growth in a context of global (i.e. social and climate) change implies adaptations to secure future feed demand and food supply (Foulkes *et al.*, 2011). Hence, the most direct solution to meet this challenge would be to increase productivity through the use of new cultivars with enhanced genetic yield potential. Wheat (*Triticum aestivum* L.) is one of the main staple crops. One of the avenues proposed to increase yield potential and improve adaptation to abiotic stresses, such as drought, is to select for higher ear photosynthesis (Tambussi *et al.*, 2005, 2007; Parry *et al.*, 2011). Indeed, the ear in wheat and other small-grain cereals is believed to play a significant role as a source of photoassimilates during grain filling, not only under drought or other abiotic stresses but also under good agronomical conditions (Araus *et al.*, 1993; Bort *et al.*, 1994; Tambussi *et al.*, 2005, 2007; Maydup *et al.*, 2010) or as response to different fungal diseases that may affect leaves (Robert *et al.*, 2005) more than ears (Tiedemann and Firsching, 2000).

Although several studies have analysed ear photosynthesis (Araus *et al.*, 1993; Bort *et al.*, 1994; Tambussi *et al.*, 2007; Maydup *et al.*, 2010), its contribution to grain filling remains unclear. The reported contributions to grain filling vary widely, with estimates ranging from about 10 to 76% of the assimilate being deposited in grains (Gebbing and Schnyder, 2001; Tambussi *et al.*, 2007; Aranjuelo *et al.*, 2011). The variability of these estimates may reflect genetic diversity in the contribution of ear photosynthesis to grain yield combined with different growing conditions, but this is also likely to be the consequence of drawbacks in the methods used. In fact, compared with the leaves, the photosynthetic contribution of the ear has been less studied, in part due to methodological limitations. In addition, the genotypic differences in the contribution of ear photosynthesis to grain filling cannot be accounted for solely on the basis of the net photosynthesis of the ears. Differences in the refixation rate of the ears could also be involved (Tambussi *et al.*, 2007). Indeed, a substantial refixation of respiratory CO_2_ within the ear has been reported (Bort *et al.*, 1996), which can contribute to 70% of the sucrose accumulated in bracts (Gebbing and Schnyder, 2001). While gas-exchange measurements are time-consuming, and even more so if respiration also needs to be monitored, there is no proven link between whole-ear photosynthesis and the relative contribution of this organ during grain filling. This is why an array of alternative approaches has been deployed for a large-scale evaluation of the ear contribution to grain filling.

Most of the methods for inferring the photosynthetic contribution of different plant parts to filling grains have involved intrusive approaches based on a differential (i.e. organ-specific) prevention of photosynthesis of some parts of the plant. Such approaches include, for example, shading the ears, the flag leaf blade, or the entire shoot (Aggarwal *et al.*, 1990; Araus *et al.*, 1993; Peralta *et al.*, 2011), application of herbicides that prevent photosynthesis (Maydup *et al.*, 2010), or simply defoliating leaf blades (Ahmadi *et al.*, 2009). Besides the intrusive nature of such treatments, it should be kept in mind that compensation effects triggered by these treatments may eventually increase the contribution of unaffected photosynthetic organs or of pre-anthesis reserves to grain filling (Aggarwal *et al.*, 1990; Eyles *et al.*, 2013).

The use of the stable carbon isotope signature in its natural abundance may help to elucidate the relative contribution of the different photosynthetic organs (Sanchez-Bragado *et al.*, 2014). The carbon isotope composition (δ^13^C; frequently expressed as carbon isotope discrimination, Δ^13^C) in plant matter reflects the photosynthetic performance of the plant (Farquhar *et al.*, 1989) and is one of the most successful time-integrated physiological traits used by plant breeders (Araus *et al.*, 2002). The stable isotope, ^13^C, is discriminated against the lighter ^12^C during photosynthetic carbon fixation (Farquhar and Richards, 1984). Thus, discrimination of ^13^C in a photosynthetic organ depends on the ratio of the intercellular versus the external (atmospheric) CO_2_ concentration of CO_2_ (*c*_i_/*c*_a_) (Farquhar *et al.*, 1989). Whereas environmental factors such as water availability may affect δ^13^C (and thus Δ^13^C), mostly through an effect on stomatal conductance, there are also constitutive differences in δ^13^C associated with the specific plant part considered (Hubick and Farquhar, 1989; Araus *et al.*, 1993). This is the case, for example, for δ^13^C from photoassimilates produced by different photosynthetic plant parts, such as the leaf blades and the ear (Hubick and Farquhar, 1989; Araus *et al.*, 1992, 1993). Thus, regardless of the growing conditions the δ^13^C of photoassimilates from the flag leaf blade is lower (more negative) than from the ear (Araus *et al.*, 1992, 1993). Such variation in δ^13^C among plant parts may be caused by differences in the *c*_i_/*c*_a_ ratio driven by a far lower permeability to gas diffusion in the ear compared with the blades. Thus, the higher constitutive δ^13^C of the assimilates from the ear compared with the leaves may be associated with a lower *c*_i_/*c*_a_ of the former organ.

The main photosynthetic organs of the ear are the glumes and the awns (Gebbing and Schnyder, 2001; Tambussi *et al.*, 2007). While in awned cereals this tissue seems to be the main photosynthetic organ of the ear in terms of fixing atmospheric CO_2_ (Li *et al.*, 2006; Tambussi *et al.*, 2007), as pointed out above, the glumes may also have a crucial photosynthetic role mainly in refixing CO_2_ respired by the forming grains (Gebbing and Schnyder, 2001).

This study proposed the use of the δ^13^C of assimilates from different plant parts as a criterion to assess in a non-disturbing manner the relative contribution of ear and shoot photosynthesis to grain filling. In such a way, the δ^13^C of assimilates from the awns and the glumes were analysed at about the mid-stage of grain filling. In order to integrate the δ^13^C of the assimilates produced by the different photosynthetic organs of the shoot and then transferred to the ear along with stem reserves, the δ^13^C of assimilates from the peduncle was also analysed. In addition, the δ^13^C of the assimilates of the flag leaf blade was also analysed, because traditionally this organ has been considered as the main photosynthetic contributor to growing grains, particularly in the absence of water stress (Evans *et al.*, 1975; Araus and Tapia, 1987). The present study was carried out on a set of high-yielding advanced lines of bread wheat from the International Maize and Wheat Improvement Center (CIMMYT), Mexico, growing under well-managed agronomic conditions.

## Material and methods

### Germplasm used and experimental conditions

Six advanced bread wheat (*T. aestivum* L.) lines were selected on the basis of their similar phenology, high grain yield, and biomass, from the CIMCOG (CIMMYT Mexico Core Germplasm) population, which is composed of 60 elite lines generated from CIMMYT breeding programmes (Table 1). The field experiments were conducted during the spring growing season of 2012 at MEXPLAT (Mexican Phenotyping Platform) situated at CIMMYT’s Experimental Station, Norman E. Borlaug (CENEB) in the Yaqui Valley, near Ciudad Obregón, Sonora, México (27°24′N, 109°56′W, 38 m above sea level), under fully irrigated conditions. The soil was a typic calciorthid with low organic matter composition (0.76%) and a slightly alkaline (7.7) pH (Sayre *et al.*, 1997) with a plant-available water-holding capacity of about 200mm (Lopes and Reynolds, 2012). The experimental design was a randomized lattice with three replications in 8.5 m long plots consisting of two raised beds (0.8 m wide per bed) with two rows per bed (0.24 m between rows) with an additional shared bed in each plot side. The seeding rates were 108kg ha^−1^. The experiments were sown on 9 December 2011 and 23 November 2012, and immediately irrigated to promote germination. The respective emergence dates were on 16 December 2011 and 2 December 2012. Harvesting was performed by machine on 15 May 2012 and manually on 6–7 May 2013, respectively, about 15–20 d after reaching physiological maturity. The mean rainfall was 14.2 and 15.4mm and evapotranspiration was 4.6 and 3.8mm, respectively, during the 2012 and 2013 crop cycles. The maximum average temperatures were 28.0 and 25.9 °C and the minimums were 8.4 and 8.3 °C (in 2012 and 2013, respectively). The relative moisture ranged from 27.5 to 88.5% in 2012 and from 34.4 to 90.9% in 2013. A total of five auxiliary irrigations were provided totalling more than 500mm of water applied in 2012 and 2013. In 2012, the auxiliary irrigation dates during grain filling were on 16 March and 31 March, about 8 and 17 d after anthesis (DAA), respectively. For the crop cycle in 2013, auxiliary irrigation dates were on 15 March and 4 April, about 8 and 28 DAA. Appropriate fertilization and weed, disease, and pest control were implemented to avoid yield limitations. Plots were fertilized with 50kg ha^−1^ of N and 50kg ha^−1^ of P at soil preparation and another 150kg ha^−1^ of N with the first irrigation.

**Table 1.** Mean values of grain yield (GY), agronomical components and phenology measured in the six selected genotypesEach value is the mean±SE of three replications. Thousand kernel weight (TKW), harvest index (HI), biomass at anthesis (BM), number of grains per spike (GSP), kernel weight per spike (KW SP^–1^), plant height (Height), and number of days from sowing to anthesis (DTA) and maturity (DTM) were determined. ANOVA for the effect of genotype is shown. Pedigrees of the genotypes detailed in the Genotype column are as follows: 1, CNO79//PF70354/MUS/3/PASTOR/4/BAV92*2/5/FH6-1-71; 2, PBW343*2/KUKUNA*2//FRTL/PIFED2; 3, SOKOLL//PBW343*2/KUKUNA/3/ATTILA/PASTOR3; 4, TACUPETO F2001/BRAMBLING*2//KACHU4; 5, UP2338*2/4/SNI/TRAP#1/3/KAUZ*2/TRAP//KAUZ/5/MILAN/KAUZ//CHIL/CHUM18/6/UP2338*2/4/SNI/TRAP#1/3/KAUZ*2/TRAP//KAUZ5; 6, WBLL1*2/KURUKU*2/5/REH/HARE//2*BCN/3/CROC_1/AE.SQUARROSA(213)//PGO/4/HUITES6. Mean values with different superscript letters were significantly different according to Tukey’s HSD test (*P*<0.05). NS, not significant.

Genotype	GY (Mg ha^−1^)	BM (Mg ha^−1^)	HI	TKW (g)	KW SP^−1^ (g)	Height (cm)	GSP	DTA (d)	DTM (d)
1	7.2±0.2^a^	16.0±0.2^b^	0.45±0.01^a^	45.5±0.1^b^	2.8±0.0^bc^	110.8±0.9^a^	61.7±0.8^a^	87^bc^	132^a^
2	6.9±0.3^a^	14.4±0.3^a^	0.48±0.03^a^	42.2±0.9^a^	2.0±0.1^a^	99.8±0.6^b^	46.9±2.9^b^	84^a^	126^a^
3	6.6±0.1^a^	15.2±0.2^ab^	0.43±0.01^a^	43.1±0.3^ab^	2.1±0.1^a^	107.4±0.9^b^	48.0±1.2^b^	86^b^	127^a^
4	6.6±0.0^a^	14.7±0.4^ab^	0.45±0.02^a^	43.0±0.8^ab^	2.7±0.1^b^	101.3±2.7^a^	61.8±2.1^a^	89^c^	131^a^
5	6.9±0.2^a^	15.7±0.6^a^	0.44±0.02^a^	40.3±0.7^a^	2.1±0.0^a^	105.1±0.7^ab^	51.3±1.1^a^	88b^c^	131^a^
6	6.6±0.3^a^	13.7±0.4^a^	0.48±0.02^a^	49.0±0.7^c^	3.1±0.1^c^	108.1±1.1^b^	63.2±2.3^b^	87^bc^	132^a^
Level of significance									
Genotype	NS	0.010*	NS	0.000***	0.000***	0.001**	0.000***	0.000***	NS

### Agronomic traits

For each plot, grain yield, biomass, yield components, and plant height were determined in approximately 5.7 m^2^ using standard protocols (Pask *et al.*, 2012). In addition, phenology was recorded throughout the cycle using the Zadocks scale (Zadocks *et al.*, 1974).

### Leaf and ear photosynthesis and respiration

Photosynthetic and respiration rates of the flag leaf blade and the ear were measured as carbon uptake using a LI-6400XT portable gas-exchange photosynthesis system (LI-COR, Lincoln, NE, USA). Photosynthesis measurements were performed approximately 2 weeks after anthesis. The flag leaf photosynthetic assimilation rate was estimated at a saturating photosynthetic photon flux density (PPFD) of 1500 μmol m^–2^ s^–1^ and 30 °C. Ear photosynthesis was measured using a hand-made chamber connected to the Li-6400XT as described previously for other purposes (Aranjuelo *et al.*, 2009). Ears were enclosed inside the chamber and ingoing air was passed through the chamber at a rate of 1 l min^–1^. The molar fractions of CO_2_ and humidity were measured with the infrared gas analyser of the LI-6400XT. The CO_2_ partial pressure was maintained as constant with the infrared gas analyser-controlled CO_2_ injection system. To ensure steady-state conditions inside the chamber, the system was left to stabilize for a few minutes. An external light source composed of LED lights was placed around the chamber during the measurement achieving a saturating PPFD of approximately 1200 μmol m^–2^ s^–1^ measured inside the chamber. The photosynthetic rates presented here are based on the whole organ area measured with a LI3050A/4 (LI-COR). Dark respiration of the flag leaf and the ear were measured immediately after the photosynthetic measurements at a temperature of 30 °C.

### Assimilates produced

The potential contribution of the two organs as a source of assimilates was assessed taking into account the instantaneous net photosynthesis plus the dark respiration (here defined as gross photosynthesis) of the whole organs multiplied by the duration of the daylight period with a saturating PPFD and the number of days from heading to organ senescence. For each organ, the active duration of the flag leaf and ear was assessed periodically from heading to maturity. In the case of the flag leaf, chlorophyll content was measured once a week with a SPAD-502 Minolta chlorophyll meter (Spectrum Technologies, Plainfield, IL, USA) in five flag leaves per plot. The active duration of the flag leaf was considered to end when SPAD values went below 20. For the ear, senescence was assessed visually and the active ear duration was considered to end when the peduncle changed colour. In addition, the total amount of assimilates produced per organ was estimated from the accumulated gross photosynthesis from heading to maturity, assuming all the fixed C was converted into carbohydrates (CH_2_O).

### Light interception

Incident and transmitted photosynthetically active radiation (PAR) was measured about 1 week after anthesis on clear days as close to solar noon as possible (from 11:00 to 14:00), with a Linear PAR Ceptometer (AccuPAR LP-80; Decagon, Washington, CD, USA). Different strata of the canopy were considered for the measurements of transmitted PAR: the base of the ear, the flag leaf blade (which also included the peduncle), the penultimate leaf (including the sheath of the flag leaf and the first internode), and the third leaf (including the sheath of the penultimate leaf and the second internode). A single measurement was performed at each stratum in each of the three replicates. The light intercepted by each stratum was estimated from the PAR measured by adapting the equations described by Pask *et al.* (2012) to each stratum.

### Total water-soluble carbohydrates (WSCs)

For the 2012 and 2013 crop seasons, WSCs were analysed in plants around mid-grain filling. Sampling was performed twice in 2012 and once in 2013. In 2012, WSCs were sampled 17 and 24 DAA (before and after irrigation, respectively). In 2013, WSCs were sampled 18 DAA. Ten representative ears, flag leaves, and peduncles per plot were harvested, cleaned, and immediately frozen with liquid nitrogen. Additionally, for the 2013 crop season, the entire peduncle was sampled and thereafter separated into two sections, the upper section (peduncle 1) and the lower sections (peduncle 2). The samples were stored at –20 °C and then lyophilized for 48h in 2012. For the 2013 crop season, samples were oven dried at 70 °C for 48h. In addition, the glumes, awns, flag leaves, and peduncles were separated, weighed, and finely ground. WSCs were analysed as described by Yem and Willis (1954) using the anthrone method and following the procedures described in Galicia *et al.* (2009). Briefly, the anthrone procedure is based on the reaction of anthrone (9,10-dihydro-9-oxoantraceno) with the furfural conformation of carbohydrates (treatment of carbohydrate in strong sulfuric acid) to give a coloured hemi-acetal, which is determined spectroscopically at 630nm. Total soluble carbohydrates are expressed on a dry-weight basis. In addition, total soluble carbohydrates per whole organ were calculated.

### Carbon isotope analysis

Analyses were only performed in the 2012 experiment. The stable carbon isotope composition (δ^13^C) in the dry matter (DM) of glumes, awns, flag leaves, and peduncles was analysed in the same samples used for WSCs and taken before irrigation (17 DAA). δ^13^C was also analysed in mature kernels. For δ^13^C analysis of the DM, approximately 1mg of each sample was weighed into tin capsules and measured with an elemental analyser coupled with an Isotope Ratio Mass Spectrometer (Delta C IRMS; ThermoFinnigan, Bremen, Germany) operating in continuous flow mode in order to determine the stable carbon (^13^C/^12^C) isotope ratios of the same samples. The ^13^C/^12^C ratios of plant material were expressed in δ notation (Coplen, 2008): δ^13^C=(^13^C/^12^C)_sample_/(^13^C/^12^C)_standard_ – 1, where ‘sample’ refers to plant material and ‘standard’ to international secondary standards of known ^13^C/^12^C ratios (IAEA CH7 polyethylene foil, IAEA CH6 sucrose and USGS 40 l-glutamic acid) calibrated against Vienna Pee Dee Belemnite calcium carbonate with an analytical precision (standard deviation) of 0.10‰.

The water-soluble fraction (WSF) of the flag leaf, peduncle, glumes, and awns was further extracted, as described previously (Yousfi *et al.*, 2013), from the same dry-matter samples used for WSCs and taken before and after irrigation. Briefly, 50mg of fine leaf and ear powder was suspended in 1ml of Milli-Q water in an Eppendorf tube (Eppendorf Scientific, Hamburg, Germany) for 20min at about 5 °C. After centrifugation (12 000*g* for 5min at 5°C), the pellet was discarded and the supernatant containing the WSF was heated at 100 °C for 3min where the heat-denatured proteins precipitated. Subsequently, samples were centrifuged again (12 000*g* for 5min at 5 °C) to separate previously denatured proteins from the soluble fraction. An aliquot of 40 µl of supernatant containing the protein-free WSF was transferred to tin capsules for carbon analysis. The capsules containing the aliquots were oven dried at 60 °C for 1h. Then, the δ^13^C of the WSCs was determined following the same procedure as that used for DM. Isotopic analyses were carried out by the Scientific-Technical Services of the University of Barcelona, Spain.

### Isotopic composition of respired CO_2_

Analysis of the isotopic composition of respired CO_2_ was performed as described previously by Nogués *et al.* (2004). Entire flag leaves and ears were placed separately in the same chamber used to measure ear photosynthesis, and this was connected in parallel to the sample air hose of a LI-6400XT (LI-COR). The measurements were performed in the field in intact plants about 2 weeks after anthesis. Measurements were done twice: during the day (covering the entire plant with a black blanket) and the subsequent night. Ingoing air was passed through the chamber at a rate of 1 l min^−1^. The CO_2_ respired by the plant was monitored by the LI-6400XT in order to determine respiration rates so that the time of accumulation could be defined to obtain a concentration in the chamber of approximately 350 ppm of CO_2_. The gas-analysis chamber was first flushed with CO_2_-free air to ensure that only the CO_2_ respired in the chamber was accumulated. According to the respiration rates, the time required for the plant to respire 350 ppm of CO_2_ was calculated and the chamber system was closed until the CO_2_ concentration inside the chamber reached the desired concentration. For each analysis, 25ml of gas sample was collected from inside the chamber with a 50ml syringe (SGE, Ringwood, Victoria, Australia) and immediately injected into a 10ml BD vacutainer. The vacutainers were sent for analysis at the Scientific-Technical Services of the University of Barcelona, Spain, and were analysed by gas chromatography combustion isotope ratio mass spectrometry as previously described (Aranjuelo *et al.*, 2009).

### Relative photosynthetic contribution to grain filling

The relative contribution to grain filling of the different photosynthetic organs of the plant was assessed by a comparison of the δ^13^C of the WSF of the different organs (averaged values before and after irrigation) and the δ^13^C of mature kernels. The approach takes into consideration several assumptions. It considers that the photosynthetic organs fixing CO_2_ from the atmosphere are the awns and the green culm parts (leaf blades, sheaths, and peduncles) and therefore it excludes the glumes because this organ mainly fixes CO_2_ from grain respiration (Gebbing and Schnyder, 2001). It assumes that the WSF in the peduncle reflects the pooled assimilates produced by the different photosynthetic organs (leaf blades, sheaths and the peduncle itself) during grain filling (plus the pre-anthesis reserves) eventually moving to growing kernels (assuming no downstream fractionation). Analysing only the WSF of the peduncle as an indicator of the pooled photosynthetic contribution of the stem is a way to economize analyses while developing a feasible technique for breeding in terms of numbers of analyses required.

The specific contribution to grain filling of the flag leaf blade was also assessed through analysis of the WSF in this organ (averaged values before and after irrigation) compared with the WSF of the awns (also averaged values), in order to estimate the potential maximum contribution of the flag leaf to grain filling. This was based on the fact that the flag leaf blade has traditionally been considered the main photosynthetic organ contributing to grain filling (Evans *et al.*, 1975; Araus and Tapia, 1987).

In addition, the approach proposed here considers that the relative contribution of each photosynthetic organ to grain filling varies as a result of water status and that it is reflected in the stable carbon isotope signature of mature grains (Araus *et al.*, 2003). In our study, relative water status was assessed through the δ^13^C of mature kernels (Farquhar and Richards 1984; Ferrio *et al.*, 2007; Araus *et al.*, 2013). Water stress may induce stomatal closure in the different photosynthetic organs and then a decrease in the ratio of intercellular to atmospheric partial pressure of CO_2_, therefore increasing the δ^13^C of assimilates (Farquhar and Richards, 1984; Condon *et al.*, 2004) and finally the δ^13^C of kernels. Thus, we assumed that the relative contribution of the awns in relation to the rest of the organs increased as water stress developed. This agrees with existing reports on the increased role of the ear (compared with the leaves) providing photoassimilates to growing kernels under water stress (Araus *et al.*, 1993; Tambussi *et al.*, 2007). Variability in crop water status may be present even under what are considered good agronomic conditions, with these frequently exposing the plants to mild water stress conditions (Cuenca, 1989).

Another assumption of the method proposed here was to neglect the δ^13^C fractionation due to translocation of assimilates from either the culm or the awns to the kernels (Yoneyama *et al.*, 1997). In fact, it has been reported that respiration associated with translocation may only have a minor discrimination effect (Bort *et al.*, 1996; Badeck *et al.*, 2005). Therefore, it was expected that the δ^13^C of the kernels would directly reflect the isotopic signal resulting from the combinations of the δ^13^C of assimilates coming from different photosynthetic sources. This implied that the same slope and origin to zero need to be found between the combined δ^13^C of the culm and the awns and the δ^13^C of the kernels.

### Statistical analysis

Data were subjected to one-way analyses of variance (ANOVA) using the general linear model in order to calculate the effects of genotype and organ on the studied parameters. Means were compared by Tukey’s honestly significant difference (HSD) test. A bivariate correlation procedure was constructed to analyse the relationships between the measured traits. Statistical analyses were performed using the SPSS 18.0 statistical package (SPSS, Chicago, IL, USA). Figures were created using the Sigma-Plot 10.0 program (SPSS).

## Results

### Effect of growing conditions on grain yield

The six selected genotypes were advanced lines that in general presented high biomass (BM) and grain yield (GY). Thus, GY across plots ranged between 6.5 and 7.2 Mg ha^–1^ (Table 1), but no significant differences across genotypes were observed. Concerning the agronomical components, thousand kernel weight (TKW) ranged from 40.3 to 49.0g and kernel weight per spike (KW SP^–1^) from 2.0 to 3.1g. All agronomic components exhibited genotypic variation except for GY and harvest index (HI). The phenology range across genotypes according to date of anthesis was 5 d, and no differences were observed for date of maturity. The duration from planting to maturity was about 130 d, whereas grain filling extended for approximately 6 weeks (counted as the number of days from anthesis to maturity).

### Photosynthetic contribution of the flag leaf and the spike to grain filling

**Fig. 1.**
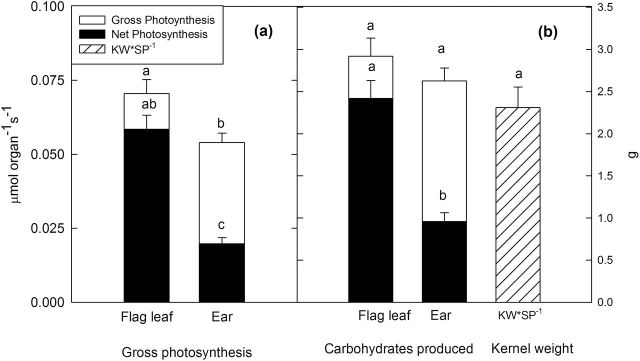
Comparative photosynthetic contribution of the ear and flag leaf during grain filling, expressed as instantaneous net photosynthetic rate plus dark respiration (gross photosynthesis) (a) and the carbohydrates produced by both organs during the reproductive stage (accounted from heading to maturity) compared with the accumulated kernel weight per spike (KW*SP^–1^) at maturity (b). Carbohydrates produced were calculated by multiplying gross photosynthesis, duration of the daylight period (at saturating PPFD), active organ duration (as the number of days from heading to maturity), and molecular weight (C_2_OH) of the basic carbohydrates produced. For more details, see Materials and methods. Each bar represents the mean values±standard error (SE) of the five genotypes with the three replications per genotype. Mean values with different superscript letters are significantly different according to Tukey’s HSD test (*P*<0.05).

Instantaneous net and gross carbon fixation were higher in the flag leaf compared with the spike (Fig. 1a). However, total photosynthetic productivities of the flag leaf and the ear (based on the accumulated gross carbon fixation) were calculated as the total carbohydrates produced by each organ from heading to maturity, and while they were comparable to the KW SP^–1^, they were not significantly different from each other (Fig. 1b).

**Fig. 2.**
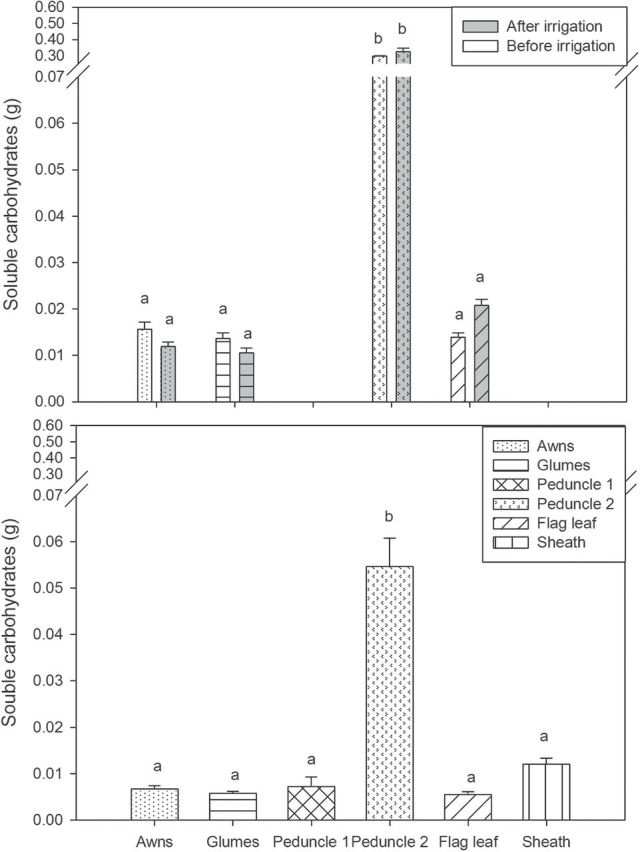
Total amount of soluble carbohydrates in 2012 (upper panel) and 2013 (lower panel) per whole organ in the awns, glumes, peduncle 1 (upper section of the peduncle), peduncle 2 (lower section of the peduncle), flag leaf, and sheath blades around mid-grain filling. Each bar represents the mean values plus standard error of the five genotypes before irrigation and after irrigation (2012) and six (2013) genotypes with three replications per genotype. The total amount of soluble carbohydrates in peduncle 1 in 2012 was calculated with the full stem weight. Mean values with different superscript letters are significantly different according to Tukey’s HSD test (*P*<0.05).

During the 2012 crop season, the amount of WSCs per whole organ present at mid-grain filling (17 and 24 DAA, before and after irrigation, respectively) in the awns, glumes, and flag leaf blades was similar, whereas in the peduncles the WSCs were significantly higher (Fig. 2, upper panel). In the 2013 crop season, the WSCs (Fig 2, lower panel) were similar in the awns, glumes, flag leaf blades and sheaths and in peduncle 1 (upper section of the peduncles). Conversely, WSCs in peduncle 2 (lower section of the peduncles) were higher than in the rest of the organs studied.

**Fig. 3.**
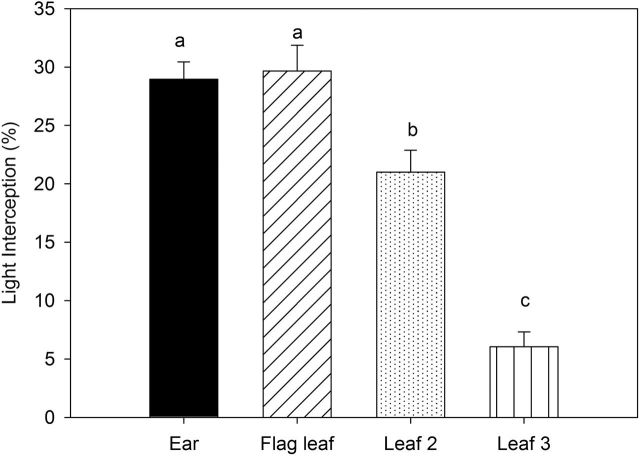
Light intercepted at different strata of the canopy: the base of the ear, flag leaf, penultimate leaf (leaf 2), and third leaf (leaf 3) around 1 week after flowering. Each bar represents the mean values±SE of the six genotypes and the three replications per genotype. Mean values with different superscript letters are significantly different according to Tukey’s HSD test (*P*<0.05).

The amount of light intercepted at the different crop strata was different among plant organs (Fig. 3). The ear and the flag leaf blade (including the peduncle) strata showed similar percentages of light intercepted (around 30%). The amount of light intercepted by the penultimate leaf (plus the first internode) was lower in comparison with that in the ear and flag leaf but higher than that of the third leaf plus the second internode.

### Carbon isotope signature

The carbon isotope composition (δ^13^C) was different between DM and the WSF, where DM showed higher values (i.e. ^13^C enriched, less negative δ^13^C) compared with the WSF before and after irrigation (Table 2). Moreover, the δ^13^C in the WSF before irrigation showed a tendency to higher values (less negative δ^13^C) compared with the WSF after irrigation, but only the peduncle showed significant differences. Significant differences in δ^13^C values were observed across plant organs, with both the DM and WSF of the awns and flag leaf blades having the highest and the lowest δ^13^C values, respectively. The δ^13^C values of the different plant organs were significantly different to the δ^13^C of mature kernels with the exception of the DM and WSF δ^13^C of the glumes. Thus, the δ^13^C of awns and peduncles exhibited slightly higher (^13^C enriched) and lower (^13^C depleted) values, respectively, than the δ^13^C of grains. Moreover, genotypic differences were found in the δ^13^C of the peduncle and flag leaf DM, whereas for the WSF only the δ^13^C in the peduncle (before and after irrigation) and the flag leaf blade (after irrigation) showed genotypic differences (see Supplementary Table S1 at *JXB* online). In spite of no significant genotypic differences in the δ^13^C_grain_, the range of variation across plots in the δ^13^C_grain_ was about 2‰. Moreover, a negative correlation across plots was observed between the δ^13^C_grain_ and GY (see Supplementary Fig. S1 at *JXB* online), which suggested that the studied trial exhibited some differences in water status across plots.

**Table 2.** Mean values and ANOVA of stable carbon isotope composition (δ^13^C) of DM and the WSF of different plant parts sampled at mid-grain filling (before and after irrigation) plus mature kernelsEach value is the mean±SE of three replications. Mean values across plant tissues with different superscripted letters are significantly different according to Tukey’s HSD test (*P*<0.05). Values with different superscript letters between brackets are significantly different between fractions and sampling dates analysed within a given organ according to Tukey’s HSD test (*P*<0.05).

	**Before irrigation**		**After irrigation**
	**δ** ^**13**^ **C DM (‰)**	**δ** ^**13**^ **C WSF (‰)**	**δ** ^**13**^ **C WSF (‰)**
Plant tissue			
Flag leaf	−28.1±0.1^a(b)^	−29.7±0.1^a(a)^	−30.1±0.1^a(a)^
Peduncle	−25.9±0.1^c(c)^	−26.9±0.1^b(b)^	−28.2±0.1^b(a)^
Glumes	−26.2±0.1^b(b)^	−26.5±0.1^c(a)^	
Awns	−25.5±0.1^d(a)^	−25.4±0.1^d(a)^	−26.1±0.1^c(a)^
Grains	−26.3±0.1^b^		
Level of significance			
Genotype	**	NS	**
Organ	***	***	***
G × O	NS	NS	NS

***P<0.001; **P<0.01; *P<0.05; NS, not significant.

### Assimilate contribution to filling grains

**Fig. 4.**
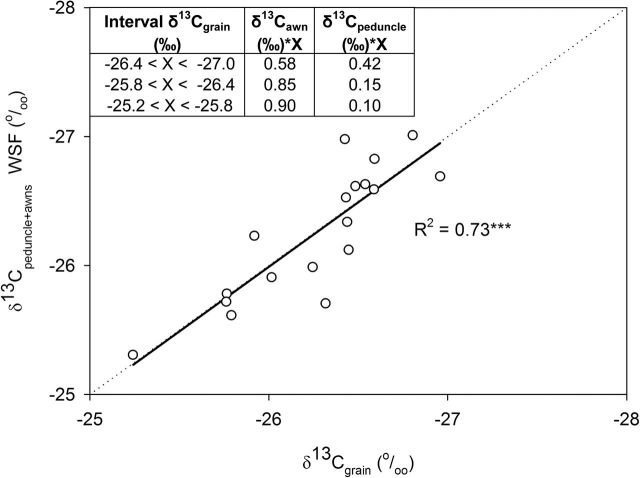
Linear regression of the relationship between the stable carbon isotope composition in mature grains (δ^13^C_grains_) and the combination of δ^13^C from awns and the peduncle (δ^13^C_awns_+δ^13^C_peduncle)_ in the WSF. The individual values of δ^13^C_awn_ and δ^13^C_peduncle_ used in the linear regression were the average of the δ^13^C in the WSF before and after irrigation. The six genotypes with three replications per genotype were considered, accounting for a total of 18 plots. For each plot, the relative weight assigned to the δ^13^C of each of the two organs depended on the water status of the plot assessed by its δ^13^C_grains_ (see inset). Level of significance: ***P<0.001.

The δ^13^C_grains_ values were between the range marked by the WSF δ^13^C of the awns (δ^13^C_awns_) and the peduncle (δ^13^C_peduncle_) at mid-grain filling (Table 2), measured before and after irrigation. The relative contribution of the δ^13^C_awns_ and δ^13^C_peduncle_ that accounted for the δ^13^C_grains_ was assessed through a linear fit. The δ^13^C_grains_ was used as a dependent variable and a combination of the δ^13^C in the WSF of awns and peduncle were used as the independent variables, with assignment of a different weight for the awn and peduncle δ^13^C depending on the water status accounted for by the δ^13^C_grains._ Thus, the δ^13^C_awns_ had a relative contribution of 90% (δ^13^C_awns_×0.90) and the peduncle 10% (δ^13^C_peduncle_×0.10) towards the δ^13^C_grain_, when the δ^13^C_grain_ values were between –25.2 and –25.8‰. Conversely, the relative contribution of the awns was 58% (δ^13^C_awns_×0.58) and the peduncle 42% (δ^13^C_peduncle_×0.42) when δ^13^C_grain_ values were between –26.4 and –27.0‰. In such a way, a linear fit with a slope of 1 and origin to zero was achieved (*R* ^2^=0.73, *P*<0.001) (Fig. 4).

**Fig. 5.**
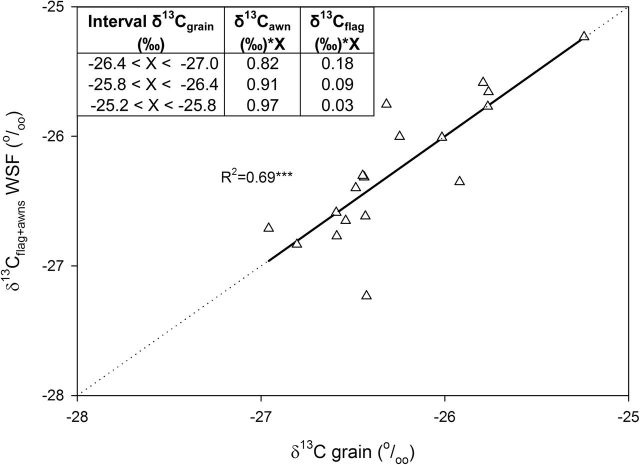
Linear regression of the relationship between stable carbon isotope composition in mature grains (δ^13^C_grains_) and the combination of the δ^13^C from the flag leaf blade and the awns (δ^13^C_flag_+δ^13^C_awns_) in the WSF. The individual values of δ^13^C_awn_ and δ^13^C_flag_ used in the linear regression were the average of the δ^13^C in the WSF before and after irrigation. The six genotypes with three replications per genotype were considered. For each plot, the relative weight assigned to the δ^13^C of each of the two organs depended on the water status of the plot assessed by its δ^13^C_grains_ (see inset). Level of significance: ***P<0.001.

The same approach was developed to assess the maximum relative contribution of the δ^13^C_flag_ to grain filling. In such a way, a combination of the WSF δ^13^C_flag_ and δ^13^C_awns_ was used as an independent variable and δ^13^C_grain_ as a dependent variable. Thus, the estimated contribution of the flag leaf was 18% (δ^13^C_flag_×0.18) and the awns 82% (δ^13^C_awns_×0.82) when the δ^13^C_grain_ values were between –26.4 and –27.0‰. By contrast, the relative contribution of the awns was 97% (δ^13^C_awns_×0.97) and the flag leaves 3% (δ^13^C_flag_×0.03) when the δ^13^C_grain_ values were between –25.2 and –25.8‰. As before, a linear fit with a slope of 1 and origin to zero was achieved (*R* ^2^=0.69, *P*<0.001) (Fig. 5).

**Fig. 6.**
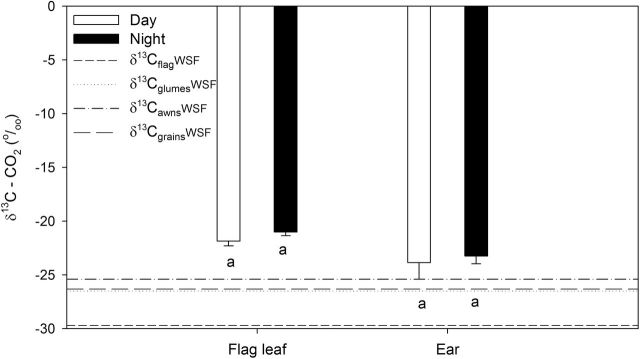
Carbon isotope composition (δ^13^C) of the CO_2_ respired by the flag leaf and the spike at mid-grain filling during the day and night (filled and open bars) compared with the δ^13^C of the WSF in the flag leaf, glumes, awns, and grains (dashed horizontal lines). Each bar represents the mean respiration. The δ^13^C values are means±SE of the six genotypes with three replicates per genotype. Mean values with different superscript letters are significantly different between day and night according to Tukey’s HSD test (*P*<0.05).

The δ^13^C of the CO_2_ respired by the flag leaf and the ear was higher (^13^C enriched) than the δ^13^C in the WSF of all the organs studied (Fig. 6). In fact, the δ^13^C_flag_ of the WSF exhibited values that were far more depleted than the δ^13^C of the CO_2_ respired by the flag leaf. On the other hand, the δ^13^C_glumes_ and δ^13^C_awns_ of the WSF and the δ^13^C_grains_ showed values only slightly more depleted than the δ^13^C of the CO_2_ respired by the ear.

## Discussion

**Fig. 7.**
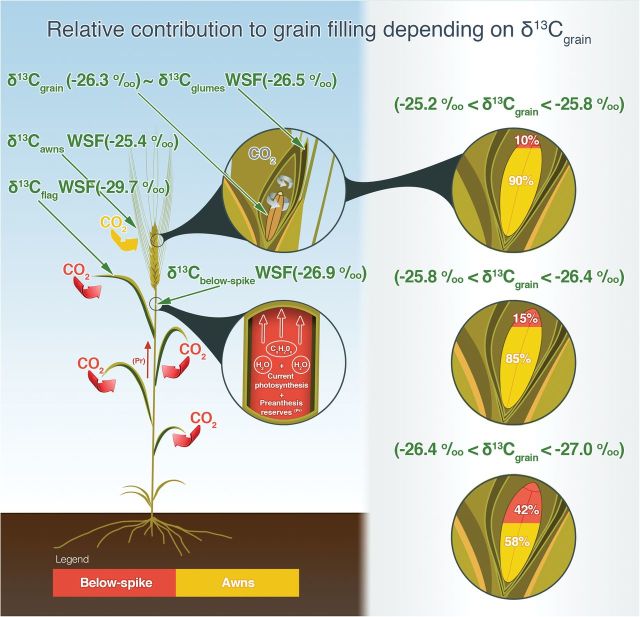
Illustration of a wheat plant showing the relative photosynthetic contributions of the ear and shoot to grain filling. The below-spike integrates the δ^13^C of the assimilates produced by the different photosynthetic organs of the shoot plus the stem reserves. The δ^13^C_awns_, δ^13^C_glumes_, and δ^13^C_below-spike_ represent the carbon isotopic composition of the WSF in the awns, glumes, and below-spike, respectively. δ^13^C_grain_ represents the carbon isotopic composition of mature kernels. (This figure is available in colour at *JXB* online.)

Our study proposes a non-intrusive methodology to quantify the relative contribution of different organs to grain filling. The approach was based on the constitutive differences in natural abundance of carbon isotopic composition (δ^13^C) of assimilates from the different photosynthetic organs active during grain filling. This method aimed to compare the δ^13^C of these assimilates with the δ^13^C of mature kernels (Fig. 7). Since the method was applied in intact (i.e. non-manipulated) plants, the results were not biased by compensatory mechanisms. Moreover, and compared with pulse-chasing approaches, this is a relatively low-cost method that may help to elucidate the relative photosynthetic contribution to grain filling of different plant organs. Further application of this methodology into breeding programmes could be considered when selection for ‘high’ spike photosynthesis is desirable (Parry *et al.*, 2011).

In order to develop a feasible technique for breeding and keeping a balance between economy and accuracy, only a few photosynthetic organs were considered. From the available literature, there is evidence that the flag leaf blade and the ear are considered the main photosynthetic organs that contribute to grain filling (Evans *et al.*, 1975; Araus and Tapia, 1987; Araus *et al.*, 1993; Bort *et al.*, 1994; Tambussi *et al.*, 2007; Maydup *et al.*, 2010). Thus, the awns and glumes, which are the two main photosynthetic parts of the ear (Bort *et al.*, 1994) were analysed separately. In addition, the δ^13^C of the assimilates was also analysed in the peduncle because this organ represents the pathway through which the current shoot assimilates (i.e. produced by the different shoot organs during grain filling, including the blades and sheaths of the flag and the lower leaves) plus the pre-anthesis reserves (assimilates accumulated before flowering) are mobilized towards growing kernels (Gebbing *et al.*, 1999). Because the use of the pre-anthesis reserves is reported to take place during the first the half of grain filling (Wardlaw and Willenbrink, 1994; Gebbing *et al.*, 1999; Zhou *et al.*, 2009), assimilates present in the peduncle at the time that samples were collected in our study may integrate the potential contribution of such reserves. Therefore, the δ^13^C in the peduncle informs us about the relative contribution of the entire culm to the grains (see Fig. 7). In any case, the potential contribution of pre-anthesis reserves to growing kernels seems at first small because the plants were grown under good agronomical conditions and so the photosynthetic capacity of the plants during grain filling exceeds the sink demand of growing grains (Slafer and Andrade, 1991; Bingham *et al.*, 2007; Dreccer *et al.*, 2009).

A basic point of our approach was that water-soluble organic matter is a proper indicator for newly produced assimilates, which agrees with available literature (Brandes *et al.*, 2006; Gessler *et al.*, 2009*b*). An additional requirement of our approach was that the δ^13^C signature in the sink was the direct consequence of the δ^13^C of assimilates produced by photosynthetic organs. Post-carboxylation fractionation effects in the δ^13^C of the newly assimilated compounds in photosynthetic organs and further fractionation during their remobilization (i.e. phloem loading, unloading, or transport) to heterotrophic tissues have been reported mostly in tree species (Damesin and Lelarge, 2003; Scartazza *et al.*, 2004; Brandes *et al.*, 2006; Bowling *et al.*, 2008; Gessler *et al.*, 2009*a*) and other woody species (Tcherkez *et al.*, 2004; Badeck *et al.*, 2005). Nevertheless, for herbaceous plants, such post-photosynthetic fractionation appears less evident. Indeed, a lack of a clear diel variation in δ^13^C in the organic WSF has been reported in sunflower (Ghashghaie *et al.*, 2001) and wheat (Kodama *et al.*, 2011). In the same sense, Yoneyama *et al.* (1997) studied post-photosynthetic fractionation of stable carbon isotopes between plant organs in different species. In their study, sugars in flag leaf blades, petioles, major veins, and phloem (which transport assimilates from source to sink tissues) of wheat were compared. Non-significant differences were found between phloem exudates (–29.5‰), sugars, and organic acids (–29.4‰) in leaf blades during grain filling. Similarly, Badeck *et al.* (2005) did not show consistent isotopic differences in the δ^13^C of sugars in the leaf blades, petioles, and major phloem veins of French bean concluding that fractionation during assimilate transport, leading to preferential export of heavy carbon isotopes from photosynthesizing leaves, cannot be proven. For herbaceous plants at least, the existence of fractionation during assimilate transport from leaves to sink tissues could not be confirmed from these results. In addition, Gessler *et al.* (2009*b*) did not find differences in δ^13^C composition of the phloem compared with the WSF and the assimilates of the leaf in *Ricinus communis*. Moreover, in our study, the CO_2_ respired in the different organs was ^13^C enriched compared with the corresponding soluble fractions (Fig. 6). This pattern of enrichment of the respired CO_2_ has been reported before in different plants (Klumpp *et al.*, 2005; Ocheltree and Marshall, 2004; Gessler *et al.*, 2009*b*), including wheat (Kodama *et al.*, 2011), and does not support an enrichment of the remaining assimilates that may eventually be translocated to the sink tissues. Such differences between the δ^13^C of the WSF (more negative) and the respired CO_2_ (less negative) were more evident in the leaves compared with other parts of the plants, which agrees with previous studies (Ocheltree and Marshall, 2004; Klumpp *et al.*, 2005).

In our study, the δ^13^C of the flag leaf was lower (more negative) than that of the ear parts, whereas the δ^13^C of mature kernels exhibited values in between. Previous studies in durum wheat (Araus *et al.*, 1993) and other cereals (Hubick and Farquhar, 1989; Araus *et al.*, 1992) have found similar patterns of lower δ^13^C in the DM and the WSF of the flag leaf in comparison with the different ear parts, while the mature kernels exhibited values between them but closer to the ear parts. Differences in organ permeability to atmospheric CO_2_ between photosynthetic organs probably explain the constitutive differences in δ^13^C of the ear compared with the flag leaf (Farquhar *et al.*, 1989; Araus *et al.*, 1993). The fact that the δ^13^C values in the WSF of the awns are far closer to the δ^13^C of mature kernels than the WSF the δ^13^C of the flag leaf supports the idea that in our study the ear has a more important role in providing assimilates during grain filling than the flag leaf. On the other hand, the values of δ^13^C in the peduncle were far higher (less negative) than those in the flag leaf, and closer (even when still more negative) to the δ^13^C of the kernel. These results provide empirical evidence that the contribution of the flag leaf to the growing grains is minor compared with the rest of the shoot (including pre-anthesis reserves).

Our results showed that the contribution of the ear represented on average about 70% of the total assimilates contributing to grain filling (Fig. 4), while the role of the flag leaf blade was markedly smaller, with an average contribution of 10% (Fig. 5). Some evidence in the past has shown that only 49% of carbon assimilated by the flag leaf moves to the grain in comparison with 80% of the ear-derived photosynthates (Carr and Wardlaw, 1965). In an experiment carried out by Aranjuelo *et al.* (2011) in durum wheat, the C fixed by the flag leaf during the beginning of post-anthesis was studied using ^13^C labelling. In this study, only a small amount of the soluble sugars coming from the C fixed by the leaf arrived at the ear, and the rest was stored as structural C compounds and starch and then respired. This study also concluded that the C synthesized in the ear was used for grain filling.

The potential contribution of the ear during grain filling is also supported by other indirect evidence. For example, the calculated total CO_2_ fixed by the ear (including the respiratory losses) was comparable to that of the flag leaf blade and of similar magnitude to the total kernel weight per spike attained at maturity (Fig. 1B) from heading to maturity. In addition, the total WSCs per whole organ at mid-grain filling, which represents the potential amount of assimilates available in this organ, also supports this assumption. Thus, the WSC values were similar in the awns, glumes, flag leaf blades (Fig. 2, upper panel) and peduncle 1 and sheaths (Fig. 2, lower panel) despite the fact that these values were approximately one sixth of the level recorded in peduncle 2 (Fig. 2, lower panel). In this sense, grain filling may be limited by the sink rather than the source in wheat (Slafer and Savin, 1994), especially under good agronomical conditions, and therefore only the assimilates from the upper part of the plant are needed to fill the grains.

Moreover, the percentage of incoming light intercepted by the ear was similar to that captured by the flag leaf blade (plus the peduncle), whereas light absorption by the rest of the shoot still accounted for about 40% of the total incoming light (Fig. 3). These results indicate that the ear may have a photosynthetic contribution during grain filling that is at least similar to that of the flag leaf, with the additional advantage that the structures of the ear are physically closer than the flag leaf to the growing kernels (Evans *et al.*, 1975). They also provide indirect evidence supporting the fact that the flag leaf blade is not the only source of assimilates from the shoot. In a study performed in durum wheat under well-watered conditions, the photosynthetic rate of the whole ear correlated much better with GY than the photosynthetic rate of the whole flag leaf blade (Abbad *et al.*, 2004). On the other hand, the relative contribution of the δ^13^C in the awns against the δ^13^C of the stem (peduncle) varied depending on the water status (see Fig. 7). The results indicate that the awns had a higher contribution to filling grains compared with the stem, especially when the water status was less optimal (i.e. less negative δ^13^C_grains_). Indeed, it has been reported that the ear is a photosynthetic organ better adapted than the flag leaf to water stress (Tambussi *et al.*, 2005). In a study carried out by Motzo and Giunta (2002) in durum wheat under Mediterranean conditions, it was concluded that the presence of awns increased the average GY by 10–16%. The positive role of awns may be even higher under drought stress. A study performed by Evans *et al.* (1972) using ^14^CO_2_ labelling revealed that the presence of awns doubled the net photosynthesis rate in the ear, and the proportion of assimilate contributed by ear photosynthesis to grain filling was greater in awned ears compared with awnless ears under drought conditions.

Our results indicated that total shoot photosynthesis (i.e. combining the contribution of the different leaves plus the peduncle together with the pre-anthesis stem reserves) represents on average 22% of total assimilates going to the grain, and up to 42% of the assimilates during grain filling when water conditions were the most optimal (and thus δ^13^C_grains_ the most negative). In addition, in order to assess which contribution of assimilates of the peduncle was actually due to the flag leaf, the maximum relative contribution of the δ^13^C in the flag leaf compared with the awns was analysed (Fig. 5). The maximum relative contribution achieved by the flag leaf was 18% (when the water conditions were the most optimal and thus the δ^13^C_grains_ the most negative). In addition, from Fig. 5, the relative contribution of the flag leaf blade appeared to be five times lower than that of the awns when water conditions were the most optimal (and thus the δ^13^C_grains_ was the most negative). Extrapolating this proportion to Fig. 4, the contribution of the flag leaf with respect to the awns was 13%. If this calculation is applied to all three water conditions, the contribution of the flag leaf with respect to the awns ranged from 3 to 13%, from less optimal to most optimal conditions, respectively. In summary, this indicates that the flag leaf blade contributes on average only 8% of grain C and that the proportion changes with the degree of water stress (experienced in this study). Moreover, the proportion of grain C coming from the below-spike photosynthetic organs other than the flag leaf blade also decreases as water stress increases. Moreover, genotypes showing higher ear contributions do not necessarily reflect higher GY. In fact, water stress (assessed by δ^13^C_grains_) may cause a decrease in GY, and thus an increase in the relative contribution of the ear to filling grains in comparison with non-ear organs (Tambussi *et al.*, 2007).

Furthermore, the glumes, which are photosynthetically active, are believed to be a significant source of assimilates for grain filling in wheat and other cereals (Araus *et al.*, 1993; Bort *et al.*, 1994). Hence, the importance of the ear’s contribution to grain filling may thus be underestimated because, in the approach presented here, the glumes were not included (Fig. 7). The glumes mainly refix CO_2_ (Bort *et al.*, 1996). In a study performed by Gebbing and Schnyder (2001) with labelling of the atmospheric CO_2_ surrounding the ear, the view was supported that the CO_2_ used for glume photosynthesis was derived mainly from CO_2_ respired by the grains. In addition, this view is reinforced by our results where the δ^13^C in the glumes and the grains was not significantly different (Table 2). In fact, no discrimination occurs during reassimilation of CO_2_ respired by the grain if the ear parts are completely gas tight (Farquhar *et al.*, 1989). These findings suggest that the ear could potentially contribute more to grain filling as was initially postulated in the approach.

Summarizing, it is not only the flag leaf that plays an important role in grain filling, as has traditionally been considered (Evans *et al.*, 1975; Araus and Tapia, 1987); the ear is also an essential organ during grain development. In accordance with the results obtained in this study, even under good agronomical conditions, the ear may be more important than the flag leaf during grain filling. Such a conclusion is also supported by the results of WSC in the whole organs, with the flag leaf blades and sheaths showing similar values to the awns and glumes as well as the similar photosynthetic contribution per whole organ recorded during the reproductive period by the ears and flag leaf blades. Whereas awns may be the organ of the ear that is pre-eminent in fixing atmospheric CO_2_ (Motzo and Giunta, 2002), the glumes may also play a major photosynthetic role in reassimilating CO_2_ respired by the ear.

Previous studies using a simplified version of the δ^13^C approach also support a very limited role for the flag leaf blade in durum wheat under both moisture stresses and low nitrogen conditions (Sanchez-Bragado *et al.*, 2014). Our results are not in conflict with a basic role of the flag and the lower leaves in providing assimilates to the growth and development of the reproductive sink; specifically, shoot photosynthesis may determine the number of fertile florets and even further, the number of kernels and their potential size (Slafer and Savin, 1994). In addition, the flag leaf also plays an important role as a source of nitrogen that is later remobilized to the grain (Bahrani and Joo, 2010).

By analysing the δ^13^C of the WSF at the peduncle, the photosynthetic contribution of the complete shoot to growing kernels has been assessed. Therefore, our methodological approach avoids the inherent limitation of not taking into account the potential contribution of other organs such as the flag leaf sheath and the peduncle, as well as the lower parts of the shoot and the pre-anthesis reserves.

The main purpose of our study was to estimate the relative organ contribution to grain filling using a non-intrusive and relatively low-cost approach (three δ^13^C analyses per plot). While such an approach may potentially be deployed as a phenotyping tool, the relative contribution of each organ to grain filling is probably strongly affected by growing conditions. Therefore, for breeding, care should be taken to assess all genotypes under similar growing conditions, avoiding as much as possible spatial (across the trial) variability in the level of soil moisture.

## Supplementary data

Supplementary data are available at *JXB* online.

Supplementary Table S1. Mean values of the stable carbon isotope composition in the flag leaf (δ^13^Cflag), peduncle (δ^13^Cpeduncle), glumes (δ^13^Cglumes), awns (δ^13^Cawns) and mature kernels (δ^13^Cgrains) of the six genotypes of bread wheat.

Supplementary Fig. S1. Polynomial quadratic regression of the relationship between the stable carbon isotope compositions (δ^13^C) of mature grains and grain yield (GY).

Supplementary Data

## Abbreviations:

ANOVA: analysis of variance
BM: biomass
CIMMYT: International Maize and Wheat Improvement Center
DAA: days after anthesis
DM: dry matter
GY: grain yield
HI: harvest index
KW SP^–1^: kernel weight per spike
PAR: photosynthetically active radiation
PPFD: photosynthetic photon flux density
SE: standard error
TKW: thousand kernel weight
WSC: water-soluble carbohydrates
WSF: water-soluble fraction.
