# Supplementary material for: Relative contribution of shoot and ear photosynthesis to grain filling in wheat under good agronomical conditions assessed by differential organ δ13C
Source: J Exp Bot. 2014 Jul 22;65(18):5401–13. doi: 10.1093/jxb/eru298 (PMC4157716; doi:10.1093/jxb/eru298)
Supplement: Supplementary Data [file supp_65_18_5401__index.html]

Relative contribution of shoot and ear photosynthesis to grain filling in wheat under good agronomical conditions assessed by differential organ δ13C — Relative contribution of shoot and ear photosynthesis to grain filling in wheat under good agronomical conditions assessed by differential organ δ13C — Supplementary Data 

# Relative contribution of shoot and ear photosynthesis to grain filling in wheat under good agronomical conditions assessed by differential organ δ13C

## Supplementary Data

Data files

**Files in this Data Supplement:**

- Supplementary Data - Supplementary Data
