# Supplementary material for: Relative contribution of shoot and ear photosynthesis to grain filling in wheat under good agronomical conditions assessed by differential organ δ13C
Source: J Exp Bot. 2014 Jul 22;65(18):5401–13. doi: 10.1093/jxb/eru298 (PMC4157716; doi:10.1093/jxb/eru298)

Relative contribution of shoot and ear photosynthesis to grain filling in wheat assessed by differential organ $\delta^{13}C$

Rut Sanchez-Bragado[1], Gemma Molero[2], Matthew P. Reynolds[2] and Jose Luis Araus[1]

Supplementary material

Table S1. Mean values of the stable carbon isotope composition in the flag leaf ($\delta^{13}C_{flag}$), peduncle ($\delta^{13}C_{peduncle}$), glumes ($\delta^{13}C_{glumes}$), awns ($\delta^{13}C_{awns}$) and mature kernels ($\delta^{13}C_{grains}$) of the six genotypes of bread wheat. For the flag leaf, peduncle, glumes and awns, both the total dry matter (DM) and the water-soluble fraction (WSF) were analysed, before and after irrigation during growing season 2012. Analysis of variance (ANOVA) for the effect of genotype is shown. Mean values with different superscripted letters are significantly different according to the Turkey's honestly significant difference test (P<0.05).

	Maturity	Before Irrigation								After Irrigation		
**Line pedigrees**	**$\delta^{13}C_{grains}$ DM (‰)**	**$\delta^{13}C_{flag}$ DM (‰)**	**$\delta^{13}C_{peduncle}$ DM (‰)**	**$\delta^{13}C_{glumes}$ DM (‰)**	**$\delta^{13}C_{awns}$ DM (‰)**	**$\delta^{13}C_{flag}$ WSF (‰)**	**$\delta^{13}C_{peduncle}$ WSF (‰)**	**$\delta^{13}C_{glumes}$ WSF (‰)**	**$\delta^{13}C_{awns}$ WSF (‰)**	**$\delta^{13}C_{flag}$ WSF (‰)**	**$\delta^{13}C_{peduncle}$ WSF (‰)**	**$\delta^{13}C_{awns}$ WSF (‰)**
CNO79//PF70354/MUS/3/PASTOR/4/BAV92*2/5/FH6-1-71	-26.6^{a}	-28.2^{ab}	-26.1^{a}	-26.3^{a}	-25.6^{a}	-29.8^{a}	-27.6^{a}	-26.4^{a}	-25.4^{a}	-30.4^{a}	-28.7^{a}	-25.4^{a}
PBW343*2/KUKUNA*2//FRTL/PIFED2	-25.8^{a}	-28.5^{a}	-25.7^{a}	-26.1^{a}	-25.3^{a}	-30.2^{a}	-26.8^{ab}	-26.9^{a}	-25.3^{a}	-29.9^{ab}	-27.8^{b}	-25.4^{a}
SOKOLL//PBW343*2/KUKUNA/3/ATTILA/PASTOR3	-26.1^{a}	-28.3^{a}	-26.0^{a}	-26.2^{a}	-25.5^{a}	-29.4^{a}	-27.0^{ab}	-26.6^{a}	-25.4^{a}	-29.5^{b}	-28.1^{a}	-25.4^{a}
TACUPETO F2001/BRAMBLING*2//KACHU4	-26.3^{a}	-28.1^{ab}	-26.0^{a}	-26.3^{a}	-25.5^{a}	-29.3^{a}	-26.7^{ab}	-26.7^{a}	-25.3^{a}	-30.2^{a}	-28.3^{a}	-25.3^{a}
UP2338*2/4/SNI/TRAP#1/3/KAUZ*2/TRAP//KAUZ/5/MILAN/KAUZ//CHIL/CHUM18/6/UP2338*2/4/SNI/TRAP#1/3/KAUZ*2/TRAP//KAUZ5	-26.4^{a}	-28.1^{ab}	-25.8^{a}	-26.0^{a}	-25.5^{a}	-30.0^{a}	-26.8^{ab}	-26.3^{a}	-25.5^{a}	-30.4^{a}	-28.5^{a}	-25.5^{a}
WBLL1*2/KURUKU*2/5/REH/HARE//2*BCN/3/CROC_1/AE.SQUARROSA(213)//PGO/4/HUITES6	-26.3^{a}	-27.5^{b}	-25.6^{a}	-25.9^{a}	-25.4^{a}	-29.7^{a}	-26.3^{ab}	-26.0^{a}	-25.5^{a}	-29.9^{a}	-27.8^{b}	-25.4^{a}
**Level of significance**												
Genotype	ns	0.02*	0.05*	ns	ns	ns	0.05*	ns	ns	*	**	ns
Rep	ns	ns	ns	ns	ns	ns	ns	ns	ns	ns	ns	ns

***$P < 0.001$; **$P < 0.01$; *$P < 0.05$; ns not significant

Fig. S1 Polynomial quadratic regression of the relationship between the stable carbon isotope compositions ($\delta^{13}C$) of mature grains and grain yield (GY). The six genotypes and three replications per genotype were considered. Levels of significance: *, $P < 0.05$

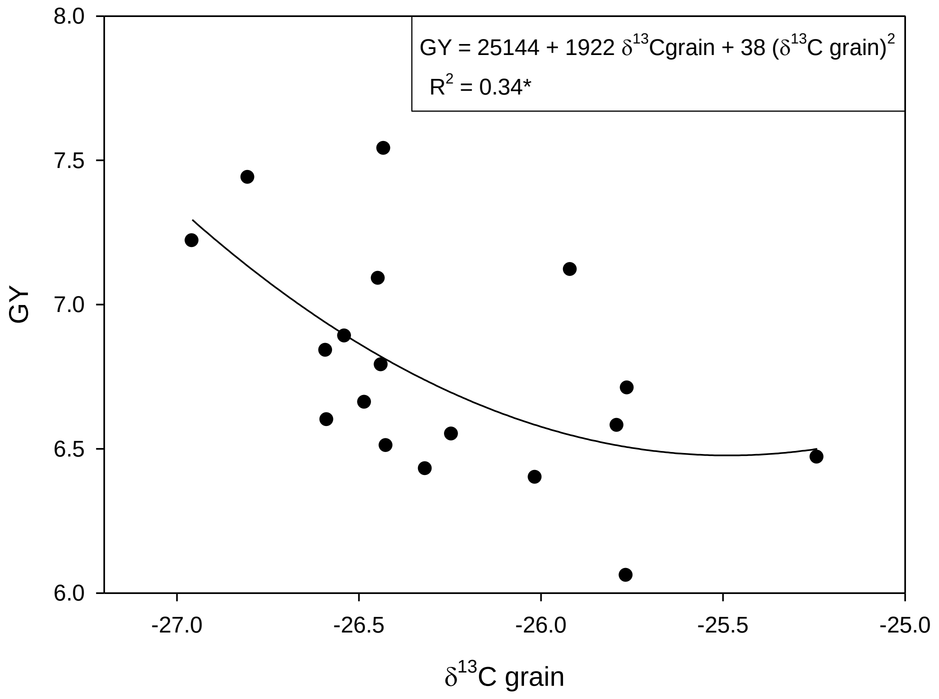

Supplement: Supplementary Data [file supp_eru298_jexbot114652_file001.pdf]
